# Subclinical atherosclerosis in psoriasis. Usefulness of femoral artery ultrasound for the diagnosis, and analysis of its relationship with insulin resistance

**DOI:** 10.1371/journal.pone.0211808

**Published:** 2019-02-08

**Authors:** Alvaro Gonzalez-Cantero, Jorge Gonzalez-Cantero, Ana Isabel Sanchez-Moya, Cristina Perez-Hortet, Salvador Arias-Santiago, Cristina Schoendorff-Ortega, Jorge Luis Gonzalez-Calvin

**Affiliations:** 1 Department of Dermatology, Complejo Hospitalario de Toledo, Toledo, Spain; 2 Department of Radiology, Gregorio Marañon Hospital, Madrid, Spain; 3 Department of Dermatology, Virgen de las Nieves Hospital, Granada, Spain; 4 Department of Gastroenterology, University Hospital San Cecilio, Granada, Spain; Medical University Innsbruck, AUSTRIA

## Abstract

**Background:**

Psoriasis is associated with an increased risk of cardiovascular disease (CVD) at younger ages that is not identifiable by traditional risk factors. Screening for subclinical atherosclerosis with ultrasound has only been investigated in carotid arteries. Femoral artery ultrasound has never been considered for this purpose. The link between psoriasis and accelerated atherosclerosis has not yet been established.

**Objective:**

To study the usefulness of femoral artery ultrasound for the detection of subclinical atherosclerosis in psoriasis. We also investigated its possible relationship with changes in insulin resistance.

**Methods:**

We conducted a cross-sectional study in 140 participants, 70 patients with moderate-to-severe psoriasis and 70 healthy controls, matched 1:1 for age, sex, and BMI. Femoral and carotid atherosclerotic plaques were evaluated by ultrasonography. Insulin resistance was assessed by the homeostasis model assessment method (HOMA-IR).

**Results:**

Femoral atherosclerotic plaque prevalence was significantly higher in patients with psoriasis (44.64%) than in controls (19.07%) (p<0.005), but no significant difference was found in carotid plaque prevalence (p<0.3). Femoral plaques were significantly more prevalent than carotid plaques (21.42%) among patients with psoriasis (p<0.001). In the regression analysis, insulin resistance was the most influential determinant of atherosclerosis in psoriasis and C-reactive protein the most significant predictor of insulin resistance.

**Conclusions:**

Ultrasound screening for femoral atherosclerotic plaques improves the detection of subclinical atherosclerosis in patients with psoriasis, whereas the study of carotid arteries is not sufficiently accurate. Insulin resistance appears to play a greater role in the development of atherosclerosis in these patients in comparison to other classical CVD risk factors.

## Introduction

Psoriasis is a complex chronic, inflammatory, immune-mediated disease of the skin and joints associated with multiple comorbidities [1, 2]. The life expectancy of patients with psoriasis is reduced by 4 to 5 years due to cardiovascular disease (CVD), and there is an increased risk of myocardial infarction at a younger age [3, 4]. It is well established that classical screening methods such as the Framingham Risk Score do not reliably evaluate the risk of coronary artery disease in patients with psoriasis [4, 5]. Early detection of subclinical coronary atherosclerosis and the adoption of primary preventive measures could minimize the risk of coronary artery disease in these patients. Rigorous screening for atherosclerosis has therefore been proposed for patients with psoriasis, emphasizing the need for a noninvasive, simple, and widely available technique for this purpose [4]. High-resolution ultrasonic arterial scanning provides information on arterial atherosclerotic plaques and meets the aforementioned criteria [6]. Carotid intima-media thickness (IMT) was initially used as a biomarker of atherosclerosis [7] but is now known to be a very weak predictor of cardiovascular risk. The IMT is not always related to atherosclerosis and does not add significant predictive capacity to traditional risk scores, and it is no longer recommended in American College of Cardiology/ American Heart Association guidelines [8–10]. A few studies used ultrasound to assess the presence of carotid plaques and reported contradictory results on their prevalence in patients with psoriasis [11–13]. Autopsy studies have observed that the presence of femoral plaque but not carotid plaque is a significant predictor of coronary atherosclerosis and coronary mortality [14, 15], and studies in healthy adults found femoral plaques to be more prevalent than carotid plaques and more strongly associated with traditional CVD risk factors and coronary calcium [16–18].

In psoriasis, there is an increased risk of myocardial infarction at a younger age that is higher than can be attributed to traditional CVD risk factors [3, 19]. This suggests that other risk factors may be implicated in the early development of atherosclerosis in these patients. Based on previous reports on a relationship between psoriasis and the increased risk of diabetes mellitus type 2 (DM-2) [20–23], we postulated that the pathogenic link between psoriasis and accelerated atherogenesis may be the presence of insulin resistance, a well-known CVD risk factor [24] that is strongly associated with psoriasis [25–27] and other chronic inflammatory conditions [28, 29].

The hypotheses of this study were that ultrasound detection of atherosclerotic plaques to screen for subclinical atherosclerosis in patients with psoriasis would be more useful in the femoral artery than in the carotid artery, and that insulin resistance may be implicated in the accelerated atherogenesis observed in these patients.

With this background, our objectives were: i) to study the usefulness of femoral *versus* carotid ultrasound for the detection of atherosclerosis in 70 patients with psoriasis and in 70 healthy controls matched 1:1 for age, sex, and BMI; and ii) to investigate changes in insulin resistance and other cardio-metabolic risk factors, analyzing their possible relationship with the presence of atherosclerotic plaques.

## Material and methods

The study was approved by the ehtics committe of Complejo Hospitalario de Toledo. All participants signed written informed consent. We conducted a cross-sectional study in 140 Caucasian participants: 70 patients with moderate to severe chronic plaque psoriasis (psoriasis area and severity index (PASI) and body surface area (BSA) values > 10), and 70 healthy control subjects matched 1:1 for age, gender, and BMI. Patients were consecutively recruited from May through September 2017 at the Department of Dermatology of our hospital in Toledo, Spain. The diagnosis of psoriasis was based on clinical findings. The other study inclusion criterion for patients was no systemic anti-psoriasis treatment for at least 3 months before the study. Exclusion criteria were: the presence of diabetes mellitus, chronic kidney disease, chronic liver disease, malignancy, chronic inflammatory disease, or arthritis, or a history of cardiovascular or cerebrovascular disease. The control group was recruited from among individuals with non- inflammatory dermatological diseases other than psoriasis (nevi, seborrheic keratosis, actinic keratosis, or verruca) and from among hospital paramedical and administrative personnel. Inclusion criteria for the controls were: age>18 years and the signing of informed consent to study participation. Exclusion criteria were the same as described above for patients plus the presence of psoriasis or a family history of this disease. Patients and controls were consecutively enrolled during the same time period, resided in the same geographic area, and signed their informed consent before study enrollment.

### Clinical and anthropometric evaluations and laboratory analysis

A full medical history was recorded, with data on mean time with psoriasis, alcohol/smoking habits, sedentarism (physical exercise < 30 min/day), and drug intake. The severity of psoriasis was quantified according to the PASI and the BSA. Waist circumference was measured with soft tape midway between the lowest rib and the iliac crest in standing position. The weight and height of participants were recorded, calculating their BMI (kg/m^2^). Blood pressure was obtained as the mean of three consecutive measurements using an OMRON M10-IT automatic oscillometric sphygmomanometer (OMRON Healthcare Co. Ltd., Kyoto, Japan), with the participant resting in seated position for 5 min between readings. Arterial hypertension was defined by a systolic blood pressure ≥140 mm Hg, diastolic blood pressure ≥90 mm Hg, or self-reported use of antihypertensive medication [30].

Blood was drawn in the morning after overnight fasting. Serum concentrations of triglycerides, high-density lipoprotein cholesterol (HDL-C), low-density lipoprotein cholesterol (LDL-C), total cholesterol, glucose, and C- reactive protein (CRP) and the erythrocyte sedimentation rate (ESR) were measured using a standard automated technique. Serum insulin was measured by electrochemiluminescence immunoassay (Elecsys 2010, Roche Diagnostics GmbH). Dyslipidemia was defined by total cholesterol ≥240 mg/dl, LDL-C ≥160 mg/dl, HDL-C <40 mg/dl, or self-reported use of lipid-lowering drugs. Diabetes was defined by fasting plasma glucose ≥ 126 mg/dl or self-reported treatment with hypoglycemic medication. Smoking was defined by current smoking status or lifetime consumption of >100 cigarettes [31].

Insulin resistance was calculated according to the homeostasis model assessment method (HOMA-IR) [32], as follows: HOMA-IR = fasting insulin (mU/L) x fasting glucose (mmol/L)/22.5. Coefficients of variation in the biochemical tests ranged from 3.1 to 9.9%. The presence of insulin resistance was defined as the highest quartile of HOMA-IR in the control group (HOMA-IR value > 2.5) as previously described [33], which is consistent with the original HOMA research report [32].

### Ultrasound study

Subjects underwent B-Mode and Doppler ultrasound examination with a MyLab 25 Gold ultrasound system (Esaote, Florence, Italy). Ultrasound images were acquired with linear high-frequency 2-dimensional probe (Esaote LA435). All participants underwent the same vascular ultrasound examination as previously described [34, 35], examining carotid arteries bilaterally in transversal and longitudinal planes from the supraclavicular fossa to the submandibular angle, including common carotid artery, carotid bulb, and origins of internal and external carotid arteries. Common femoral arteries were examined bilaterally, assessing the 20 mm segment proximal to the bifurcation in superficial and deep femoral arteries. Atherosclerotic plaque was defined as a focal structure encroaching at least 0.5 mm into the arterial lumen or having a thickness ≥ 50% of the surrounding IMT [34]. Atherosclerosis was defined by the presence of any plaque in carotid or femoral arteries. Three measurements were made of each atherosclerotic plaque thickness, calculating the mean value. A single experienced radiologist physician (JGC) performed all ultrasound examinations, blinded to the patient or control status of participants except when skin lesions were visible to the naked eye.

### Statistical analysis

Results were expressed as means ± standard deviation (SD). The Kolmogorov-Smirnoff test was used to check the normality of the data distribution. Mean values were compared among groups with one-way ANOVA, unpaired Student’s two-tailed t-test, or non-parametric Mann-Whitney U test, as appropriate. The chi-square test was used to compare proportions in different groups. Correlations were examined by Pearson standard linear regression analysis (normal distribution) or by the Spearman test (non-normal distribution). Binary logistic regression analysis was performed to establish the most significant determinants of subclinical atherosclerosis, entering: age, sex, waist circumference, BMI, HOMA-IR, physical activity level, hypertension, dyslipidemia, cigarette smoking, disease duration, PASI, BSA, and ESR values, mean time with psoriasis, and serum CRP, triglyceride, HDL-C, and LDL-C values. Binary logistic regression analysis was also used to establish the most significant determinants of insulin resistance, entering the same variables except for HOMA-IR.

Only variables with P < 0.05 were retained in the final regression model. SPSS version 22 (IBM SPSS Inc., Chicago IL) was used for data analyses.

## Results

Table 1 exhibits the anthropometrical and clinical data of participants. No significant differences were found between psoriasis patients and controls in age, sex, BMI, waist circumference, arterial hypertension, dyslipidemia, daily physical activity, or tobacco consumption.

**Table 1:** Anthropometrical and clinical data for patients with psoriasis and controls.

	Psoriasis n = 70	Controls n = 70	P<
Sex (m/f)	49/21	49/21	NS
Age (years)	45.20±11.68	43.95±11.09	NS
Body Mass Index (Kg/m2)	29.84±5.27	28.25±4.61	NS
Waist Circumference (cm)	102.11±13.32	98.92±13.91	NS
Systolic blood pressure (mm Hg)	131.02±14.54	129.38±12.72	NS
Diastolic blood pressure (mm Hg)	82.62±9.12	79.70±7.80	NS
Sedentarism (%)	12.50	16.07	NS
Smoking (%)	35.71	22.85	NS
Hypertension (%)	25.71	17.14	NS
Dyslipidemia (%)	24.28	22.85	NS
Mean time with psoriasis (years)	17.38±11.60	-	-
PASI	12.40±4.19	-	-
BSA	15.13±8.73	-	-

PASI, Psoriasis Area and Severity Index; BSA, body surface area; NS, non-significant; Data are expressed as means ± standard deviation. The chi-square test was used to compare proportions between groups.

Table 2 displays the biochemical data, showing significantly higher HOMA-IR (p<0.01), serum triglyceride (p<0.008), and CRP (p<0.01) and significantly lower HDL-C (p<0.05) in patients than in controls.

**Table 2:** Biochemical data for patients with psoriasis and controls.

	Psoriasis n = 70	Controls n = 70	P<
Glucose (mg/dl)	99.08±15.97	94.92±8.98	NS
Cholesterol (mg/dl)	190.56±32.24	193.63±37.80	NS
LDL (mg/dl)	111.94±28.48	116.43±34.16	NS
HDL (mg/dl)	50.42±13.68	56.21±14.72	0.05
Triglycerides (mg/dl)	144.10±90.16	103.06±51.47	0.008
25-OHD (ng/mL)	20.40±9.18	20.31±8.06	NS
CRP (mg/dl))	4.31±4.74	2.26±2.63	0.01
ESR (mm/h)	6.72±6.97	5.67±5.02	NS
HOMA IR	4.62±3.57	2.67±2.11	0.01

OHD, hydroxyvitamin D; CRP, C-reactive protein; ESR, erythrocyte sedimentation rate; HOMA-IR, homeostasis model assessment of insulin resistance; NS, non-significant; Data are expressed as means ± standard deviation.

### Femoral and carotid atherosclerosis

**Fig 1:**
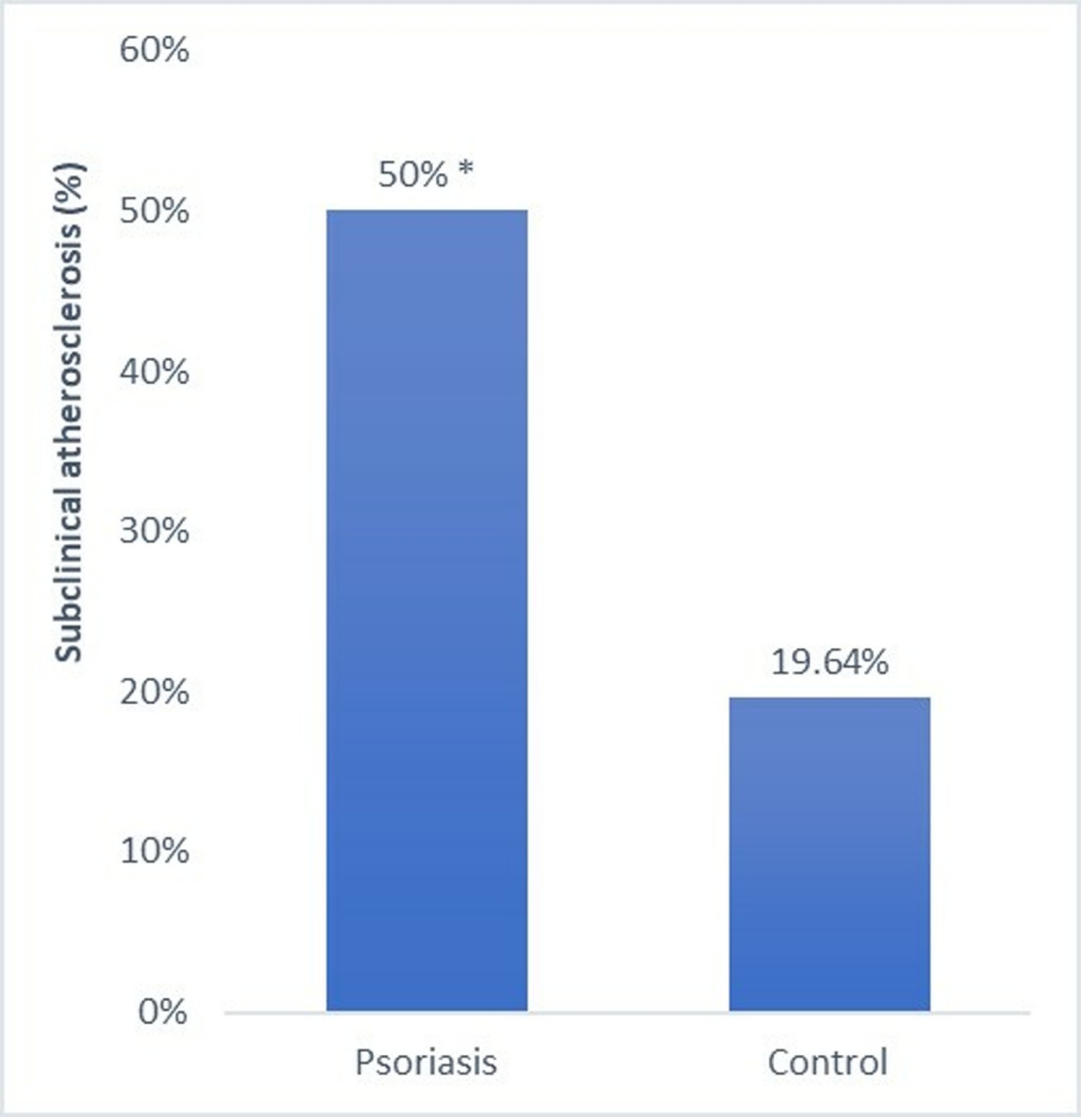
Percentage of participants with subclinical atherosclerosis among patients with psoriasis and controls. *: p< 0.05.

As previously reported in detail using a smaller sample size [36], the prevalence of atheroma plaque in both femoral and carotid arteries was significantly higher in patients with psoriasis than in controls (p<0.003) (Fig 1).

**Fig 2:**
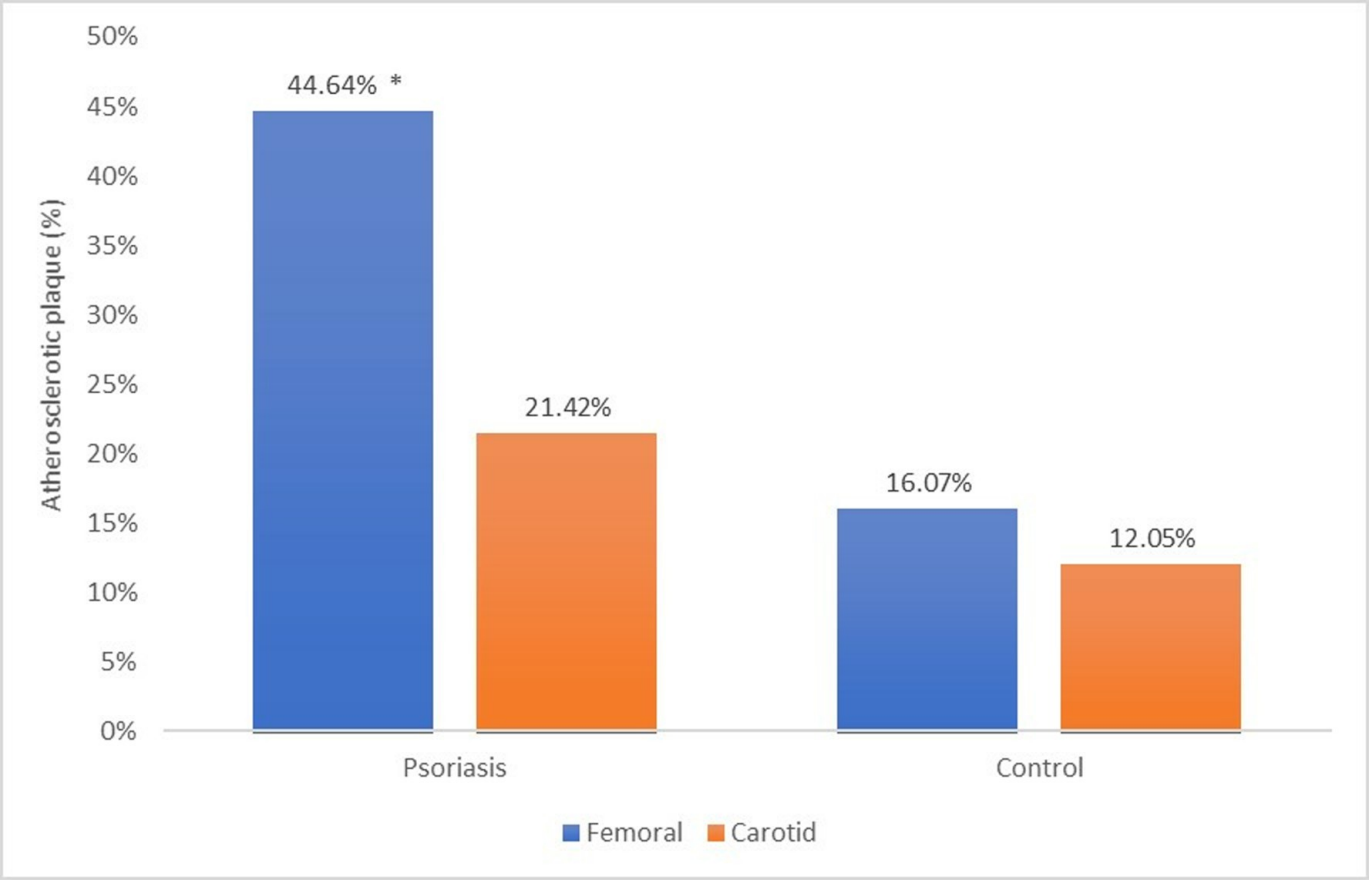
Percentage of participants with atherosclerotic plaque in femoral and carotid arteries among patients with psoriasis and controls. *: p< 0.05.

Femoral plaque prevalence was significantly higher in patients than in controls (p<0.005), as depicted in Fig 2, but no significant difference was found in carotid plaque prevalence (p<0.3). Femoral plaques were significantly more prevalent than carotid plaques among patients with psoriasis (p<0.001), whereas there was no difference in plaque prevalence between femoral and carotid arteries among controls (p<0.5).

### Relationship of anthropometric, clinical, and biochemical variables with atherosclerosis

Table 3 shows the comparison of anthropometric, clinical, and biochemical variables between patients with *versus* without atherosclerosis. As can be observed, age (p<0.001), BMI (p<0.004), waist circumference (p<0.001), hypertension (p<0.005), serum CRP concentrations (p<0.04) and HOMA-IR (p<0.004) were higher in psoriasis patients with femoral and/or carotid plaques than in those with no atherosclerotic plaque. No significant differences in sex, dyslipidemia, daily physical activity, smoking, or alcohol consumption were found between patients with *versus* without subclinical atherosclerosis.

**Table 3:** Anthropometrical, clinical and biochemical data for 70 patients with psoriasis classified according to the presence or absence of subclinical atherosclerosis.

	Psoriasis with subclinical atherosclerosis n = 35	Psoriasis without subclinical atherosclerosis n = 35	P<
Sex (m/f)	24/11	24/11	NS
Age (years)	50.69±10.29	38.52±9.70	0.001
Body Mass Index (Kg/m2)	32.31±4.92	27.80±5.55	0.004
Waist Circumference (cm)	107.92±11.70	96.60±12.68	0.001
Sedentarism (%)	15.38	12	NS
Smoking (%)	30.76	44	NS
Hypertension (%)	42.30	8	0.005
Dyslipidemia (%)	26.92	24	NS
Mean time with psoriasis (years)	18.27±10.98	14.96±9.74	NS
PASI	12.02±3.90	12.49±4.74	NS
BSA	13.85±5.17	15.28±11.29	NS
Cholesterol (mg/dl)	194.95±28.99	182.90±35.66	NS
LDL (mg/dl)	115.00±24.28	105.00±31.69	NS
HDL (mg/dl)	46.85±11.07	53.62±13.98	NS
Triglycerides (mg/dl)	173.60±113.85	120.71±61.50	NS
25-OHD (ng/ml)	17.55±8.03	20.24±6.26	NS
CRP (mg/dl)	5.39±5.31	3.37±4.55	0.04
ESR (mm/h)	8.40±7.84	6.45±6.57	NS
HOMA IR	5.68±4.01	3.57±3.29	0.004

PASI, Psoriasis Area and Severity Index; BSA, body surface area; OHD, hydroxyvitamin D; CRP, C-reactive protein; ESR, erythrocyte sedimentation rate; HOMA-IR, homeostasis model assessment of insulin resistance; NS, non-significant; Data are expressed as means ± standard deviation.

### Correlation studies

HOMA-IR was significantly and positively correlated with PCR (r: 0.58; p<0.0001), BMI (r: 0.71; p<0.0001), waist circumference (r: 0.62; p<0.0001), age (r: 0.31; p<0.04), triglycerides (r: 0.45; p<0.003), and ALT (r: 0.39; p<0.01) and was negatively correlated with HDL-C (r:0.57; p<0.0001) in the patients with psoriasis.

PCR was significantly and positively correlated with HOMA-IR (r: 0.58; p<0.0001), BMI (r: 0.51; p<0.001), waist circumference (r: 0.32; p<0.03), age (r: 0.31; p<0.04), and ESR (r: 0.34; p<0.03) in the patients with psoriasis.

### Regression analysis

Results of the stepwise logistic binary regression analysis showed that insulin resistance (OR: 7.77; 95% confidence interval (CI): 1.35–44.81; p<0.02) and age (OR: 1.14; 95% CI: 1.03–1.25; p<0.007) were the most important predictors of subclinical atherosclerosis. CRP (OR: 3.29; 95% CI: 1.13–9.56; p<0.02) was the most important predictor of insulin resistance.

## Discussion

The main finding of this study is that screening for femoral plaques improves the detection of subclinical atherosclerosis in patients with psoriasis, whereas the study of carotid arteries is not sufficiently accurate. Insulin resistance was a more influential determinant of subclinical atherosclerosis in these patients in comparison to other classical CVD risk factors, and age was also a significant predictor. The prevalence of femoral but not carotid atherosclerotic plaques was significantly higher in patients with psoriasis than in age-, sex-, and BMI-matched controls, and the patients had a two-fold higher prevalence of femoral *versus* carotid plaques.

Previous studies on the usefulness of ultrasound images to identify subclinical atherosclerosis in these patients investigated carotid arteries alone, initially by IMT examination and subsequently searching for atherosclerotic plaques. However, conflicting results were obtained, with one study finding a higher prevalence of carotid plaques in patients with psoriasis than in controls [11], whereas two observed no significant between-group difference in carotid plaque prevalence [12, 13]. Hence, there is currently no consensus on the screening test of choice to identify atherosclerosis in these patients, and the value of femoral plaque detection for this purpose has not yet been considered. In our study, unlike in the case of femoral plaques, we found no significant difference between patients and controls in the prevalence of carotid plaques, indicating that ultrasound study of the carotid artery alone is inadequate. This limitation may explain the contradictory results obtained in the aforementioned studies on carotid arteries [11–13]. According to our findings, information on femoral plaques is more useful than information on carotid plaques to identify atherosclerosis in patients with psoriasis. However, while our findings are in accordance with studies in healthy subjects [14–17] they differ from some previous studies in patients with rheumatoid arthritis (RA) or systemic lupus erythematosus (SLE), which reported a higher prevalence of carotid than femoral plaques [37–39]. We have no simple explanation for the discordant results, but it is possible that RA and SLE might have a differential impact on arterial territories due to poorly understood immunological, environmental, and/or genetic risk factors.

The pathogenesis of psoriasis-associated accelerated atherosclerosis is not clear [3]. However, our psoriatic patients showed significantly higher insulin resistance, a well-known risk factor for CVD [24] and closely associated with psoriasis [25–27]. This finding is strengthened by our exclusion of participants with DM-2 and our matching (1:1) of the controls with the patients for the main confounding factors (sex, age, and BMI). According to these results, insulin resistance in psoriatic patients appears to be at least partially independent of sex, age, and BMI and is likely associated with psoriasis *per se*. Insulin resistance, age, BMI, waist circumference, hypertension, serum CRP, and serum triglycerides values were higher in the patients with *versus* without subclinical atherosclerosis; however, insulin resistance and age were the only significant predictors of subclinical atherosclerosis in the regression analysis. These findings suggest that insulin resistance may play a more important role than that of other classical CVD risk factors in the development of atherosclerosis in patients with psoriasis.

There were no significant differences in traditional CVD risk factors between patients and controls; however, our data indicate the possible influence of comorbidities (e.g., obesity, hypertension, hyperlipidemia) and unhealthy behaviors (e.g., sedentarism, smoking) [40], given that the psoriatic patients who had subclinical atherosclerosis were older, with significantly higher BMI, waist circumference, insulin resistance, and serum CRP values, and were more frequently hypertensive in comparison to those who did not. These factors, especially insulin resistance, may therefore play a role in the etiology of atherosclerosis in these patients.

Although preliminary observations were described by our group in a letter to the editor [36], this is the first published study to evaluate the usefulness of femoral atherosclerotic plaque detection to screen for subclinical atherosclerosis in patients with psoriasis, analyzing its possible relationship with insulin resistance. Previous studies described an association between insulin resistance and endothelial function in psoriasis but reported no information on atherosclerosis [26]. We have no ready explanation for the high insulin resistance values found in our patients with psoriasis in comparison to matched control subjects, given that we controlled for age, sex, and BMI, the main insulin resistance risk factors. However, psoriasis is a systemic inflammatory disease whose pathophysiology shares several genetic [41,42] and inflammatory [43,44] abnormalities with insulin resistance. In our study, serum CRP concentrations were highly correlated with HOMA-IR values and were the only significant predictor of insulin resistance in the regression analysis, suggesting a possible role for inflammation in the development of insulin resistance in psoriasis. In addition, it has been proposed that cytokines involved in the pathogenesis of psoriasis (e.g., TNF-α and IL-17) may also affect glucose metabolism, possibly promoting insulin resistance and thereby increasing the risk of CVD in these patients [45,46]. All of these findings support previous epidemiological and prospective studies on the increased risk of DM-2 in patients with these diseases [22,47,48].

Study limitations include the relatively small sample size, although it proved possible to classify participants in different groups with adequate statistical power. Further studies are required to verify these findings in wider samples of patients and controls. Furthermore, it was not possible to investigate the natural history or clinical consequences of atherosclerosis due to the cross-sectional study design. Study strengths include the consecutive enrollment of patients; the strict exclusion criteria; the 1:1 matching of controls with patients for age, gender, and BMI; and the absence of any significant difference in traditional CVD risk factors between patients and controls.

In conclusion, ultrasound study of femoral arteries improves the detection of subclinical atherosclerosis in patients with psoriasis, whereas the study of carotid arteries is not sufficiently accurate. Insulin resistance appears to play a greater role than that of other classical CVD risk factors in the development of atherosclerosis in these patients.

Psoriasis increases insulin resistance, regardless of age, sex, or BMI, and the link between them appears to be the chronic inflammation associated with this disease.
